# Nano-Magnetic NiFe_2_O_4_ and Its Photocatalytic Oxidation of Vanillyl Alcohol—Synthesis, Characterization, and Application in the Valorization of Lignin

**DOI:** 10.3390/nano11041010

**Published:** 2021-04-15

**Authors:** Afnan Al-Hunaiti, Asma Ghazzy, Nuha Sweidan, Qassem Mohaidat, Ibrahim Bsoul, Sami Mahmood, Tareq Hussein

**Affiliations:** 1 Department of Chemistry, The University of Jordan, Amman 11942, Jordan; a.alhunaiti@ju.edu.jo; 2 Department of Chemistry, Al-Ahliyya Amman University, Amman 19328, Jordan; a.alghazzy@ammanu.edu.jo; 3 Department of Chemistry, University of Petra, Amman 11196, Jordan; nswweidan@uop.edu.jo; 4 Department of Physics, Yarmouk University, Irbid 21163, Jordan; q.muhaidat@yu.edu.jo; 5 Department of Physics, Al Al-Bayt University, Mafraq 13040, Jordan; ibrahimbsoul@yahoo.com; 6 Department of Physics, The University of Jordan, Amman 11942, Jordan; s.mahmood@ju.edu.jo; 7 Department of Physics and Astronomy, Michigan State University, East Lansing, MI 48824, USA; 8 Institute for Atmospheric and Earth System Research (INAR/Physics), University of Helsinki, FI-00014 Helsinki, Finland

**Keywords:** synthesis, characterization, aerobic, TEMPO, green catalyst

## Abstract

Here, we report on a phyto-mediated bimetallic (NiFe_2_O_4_) preparation using a *Boswellia carterii* extract, which was characterized by XRD, FT-IR, TGA, electron microscopy, magnetic spectroscopy, and Mössbauer spectroscopy measurements. The prepared nano-catalysts were tested for oxidation of lignin monomer molecules—vanillyl alcohol and cinnamyl alcohol. In comparison with previously reported methods, the nano NiFe_2_O_4_ catalysts showed high photocatalytic activity and selectivity, under visible light irradiation with a nitroxy radical initiator (2,2,6,6-tetramethylpiperidinyloxy or 2,2,6,6-tetramethylpiperidine 1-oxyl; TEMPO) at room temperature and aerobic conditions. The multifold advantages of the catalyst both in terms of reduced catalyst loading and ambient temperature conditions were manifested by higher conversion of the starting material.

## 1. Introduction

The limited reserves of traditional energy sources, coupled with the global warming trend, have created a high demand for finding alternative renewable energy sources, and sustainable, environmentally-friendly processes [1,2]. In this regard, valorization of renewable lignocellulosic biomass, most especially lignin, is a promising approach with which to produce important chemicals and fuels from available low-cost renewable materials [3,4,5,6,7]. Previously, valuable products were obtained from lignin derivatives using a variety of approaches, including hydro-processing, pyrolysis, and oxidation [8,9,10,11,12]. Specifically, important platform chemicals with improved functionality were derived from lignin by oxidation [10,11,12,13,14,15,16,17]. Photocatalysis has several environmental implications [16,17]. Photocatalytic approaches are designed to be sustainable; they should be environmentally friendly, and employ efficient light irradiation technologies for biomass valorization with degradable pollutants and the production of essential biofuels [18,19,20,21].

Over the past few years, nano-catalysts, in particular, transition metal oxide nanoparticles, have drawn substantial attention due to their higher catalytic activity compared to metallic nanoparticles [22]. For example, stable and easily synthesized NiFe_2_O_4_-NPs have been widely applied as catalysts in nanomaterial synthesis and adsorption, photocatalysis, and solar cells [23,24,25,26,27,28,29,30,31]. In addition, owing to their magnetic nature, NiFe_2_O_4_-NP catalysts are easily separable from a reaction by an external magnet [32,33]. Photocatalytic oxidation had emerged as a sustainable and viable pathway for the active catalytic species generated from the interaction of catalyst with oxygen containing oxidant by irradiation with light under environmentally-friendly reaction conditions [34]. The spinel NiFe_2_O_4_-NP has various advantages, such as low cost, good visible light response, and photochemical stability. Therefore, it can be used in solar cell applications and in photocatalytic oxidation [34]. Accordingly, the synthesis of NiFe_2_O_4_ nanoparticles with controlled size and morphology is of considerable interest. A phyto-organic extract synthesis of nanoparticles as reducing agents had been widely used lately [35,36,37].

Since lignin’s structure is complicated, most of its chemical valorization studies have been performed using model compounds. The most representative lignin model compounds are the aromatic monomers present in the lignin structure, such as guaiacol, vanillyl alcohol (VAL), and veratryl alcohol [12,38,39,40]. Both homogeneous and heterogeneous systems have been used for the oxidation of lignin model compounds. Vanillin (VN) (4-hydroxy-3-methoxybenzaldehyde) has valuable applications as a flavoring molecule [41].

Although vanillin is conventionally extracted from *Vanilla planifolia*, the high demand on vanillin led the chemical synthesis routes to be the dominant sources of vanillin. Recently, the use of petrochemical raw material, guaiacol, was recognized as a popular method for producing vanillin through multiple steps [42,43,44]. Although some researchers succeeded in obtaining VN from vanillyl alcohol (VAL) via oxidation, many of their methods required large quantities of an oxidant such as H_2_O_2_ [45,46,47,48]. Therefore, oxidative catalytic reactions using oxygen gas (O_2_) as a direct oxidant emerged as interesting approaches to convert VAL into VN [47,48]. Therefore, taking all the challenges into account, we report that NiFe_2_O_4_ nanoparticles were phyto-mediated synthesized using an aqueous extract of *Boswellia carterii* resin, which acted as a reducing agent in the synthesis of the nanoferrite. *Boswellia carterii* is of the family *Burseraceae*, the extract of which contains boswellic acid and other acids which can be used as reducing agents [47,48]. The synthesized NPs were in turn used as aerobic photocatalysts along with TEMPO for the selective oxidation of (VAL) into vanillin under environmentally-friendly conditions. To the best of our knowledge, there are no previous studies reporting on visible-light-induced photocatalysis of NiFe_2_O_4_ in the lignin substrate model oxidation under aerobic conditions.

## 2. Materials and Methods

### 2.1. Chemicals, Solvents, and Starting Materials

Iron (III) chloride anhydrous, nickel acetate, and all solvents were purchased from Sigma-Aldrich (Taufkirchen, Germany) and used as received. The crystallographic structure of MFe_2_O_4_ nano-ferrites was determined from the powder X-ray diffraction (P-XRD) pattern, whereas the particle morphology and size distribution were determined with the aid of electron microscopy imaging. PANalytical’s X’Pert PRO diffractometer with a mnochromatic Cu-K_α_ radiation (*λ* = 0.15406 nm) was employed to collect the diffraction pattern over the angular range 20° ≤ 2θ ≤ 100° in steps of 0.02°. On the other hand, field emission scanning electron microscopy (FESEM) and transmission electron microscopy (TEM) images of the nanoferrite powder were obtained using FEI QUANTA 200 (SEM, Akishima, Japan) and JEOL 6400 (SEM, Akishima, Japan), respectively. The TGA curve of the synthesized nanoferrite was determined using 40 mg sample and a rate of heating of 10 °C per min. GC–MS analysis was done for the extracted crude using a Varian 2200 GC–MS system (Saturn 2200, Varian Inc., LabX, Mid-land, ON, Canada) equipped with FID detector and RTx-5ms capillary column (30 m × 0.25 mm with maximum temperature of 350 °C). Helium (99.999% purity) was used as a carrier gas at a constant flow rate. The injected sample volume was 1 mL, with a 40:1 split ratio. The oven temperature was increased at 3 °C/min from 60 °C to 240 °C.

### 2.2. Preparation of the Plant Extract

The procedure of preparing the plant extract was described previously [49,50,51,52,53]. The *Boswellia carterii* resin was washed several times, and 200 g of plant segments were ground and mixed with 200 mL distilled water, and subsequently the mixture was refluxed at 90 °C for 3 h. The filtered extract was then used for the synthesis of the ferrite nanoparticles. The mass spectra of the sample were used to identify the existing compounds by comparison with the mass spectrum libraries (NIST 2011 v.2.3 and Wiley, 9th edition).

### 2.3. Synthesis and Characterization of NiFe_2_O_4_ Nanoparticles

The nanoferrite was prepared via co-precipitation mechanism using the aqueous extract with a mixture of 1 mM Nickel nitrate Ni(NO_3_)_2_.H_2_O and 1 mM iron (III) chloride (FeCl_3_) solutions (1:1 *v*/*v*). The mixture was left for 1 h, and then NaOH solution was used to adjust the pH of the mixture to 10–12. The resulting solution was centrifuged and the solid was separated. Subsequently, the sample was dried in an oven at 80 °C for 4 h, washed with water, and then calcinated at 800 °C to form the desired NiFe_2_O_4_ spinel phase via solid state reaction mechanism. The crystal structure of the prepared nanoparticles was examined by XRD analysis. The magnetic measurements at room temperature were carried out using a commercial vibrating sample magnetometer (VSM; MicroMag 3900, Princeton Measurements Corporation, Princeton, NJ, USA). 

### 2.4. Mössbauer Spectroscopy

The Mössbauer spectrum of the synthesized NiFe_2_O_4_ nanoferrite was measured by a conventional ^57^Fe constant acceleration Mössbauer spectrometer equipped with a ^57^Co/Rh source. The sample was prepared from the ferrite powder in the shape of a 2-cm diameter thin circular layer pressed between two Teflon disks. The Mössbauer spectrum was recorded over 1024 channels, and calibrated using room temperature iron metal spectrum. The spectrum of the ferrite was fitted with two magnetic sextet components using least-squares-base fitting routines, and the hyperfine parameters were determined from the results of the fit.

### 2.5. Photocatalytic Reaction

The prepared NiFe_2_O_4_-NPs were used in the oxidation of VAL. In total, 1 mmol of vanillyl alcohol, 0.0207 mmol (20 mg) of the magnetic NiFe_2_O_4_-NP, 2 mmol of NH_4_Br, and air bubbling were added to microphotoreactor under a 7 W LED. Moreover, there were two ports for inserting a syringe for the purpose of sampling and for introducing oxygen as an oxidant. Before turning the lamp on, and in order to reach the adsorption–desorption equilibrium, the reaction mixture was stirred for 30 min in the dark. After the required time interval of irradiation, the catalyst was removed by a magnet, and the samples were taken for analysis. The samples were analyzed by GC–MS and ^1^H-NMR and ^13^C-NMR and compared to the commercially available chemicals.

### 2.6. Experimental Procedure for Reusing of the Catalyst

The reaction was repeated with vanillyl alcohol as a substrate in 1.0 mmol scale while keeping the reaction conditions constant, except for the use of the recycled NiFe_2_O_4_ catalyst rather than fresh catalyst. After the end of reaction, the catalyst was removed using an external magnet. After that, the catalyst was washed with water (5 mL) and ethanol (5 mL) two times. Finally, the resulting solid residue (NiFe_2_O_4_-NP) was oven dried. The dried catalyst was reused for further catalytic cycles up to 4 times without drastic loss of reactivity.

## 3. Results and Discussion

### 3.1. Structural Analysis

The structure of spinel ferrite (MeFe_2_O_4_; Me is a divalent ion) is cubic, where the Me^2+^ and Fe^3+^ metal ions occupy the tetrahedral (A) site (one ion per molecule), and the octahedral (B) site (two ions per molecule). The magnetic structure of the spinel ferrite is determined by the superexchange (antiferromagnetic) interactions between the magnetic ions at these sites. Since the A–B interactions between ions at different sites are significantly stronger than the interactions between ions at similar sites (A–A and B–B interactions), the magnetic ions at different sites couple antiferromagnetically, resulting in spin-down (A) and spin-up (B) sublattices [54,55]. Given that the number of ions at octahedral sites (sin-up) is more than the number at tetrahedral sites (spin-down sites), the spinel ferrite is generally ferrimagnetic, with a net magnetic moment resulting from the difference between the magnetic moments of the two sublattices. Accordingly, the ferrite shows a ferromagnetic-like behavior. Normally, nickel ferrite (NiFe_2_O_4_) is an inverse spinel, where the Ni^2+^ ions occupy the octahedral (B) sites, whereas half of the Fe^3+^ ions occupy octahedral sites and the other the other half the tetrahedral (A) sites. In this case, the magnetization is determined by magnetic moment of the Ni^2+^ ion, since the magnetic moments of the Fe^3+^ ions at octahedral and tetrahedral sites cancel out. Accordingly, nickel bulk ferrite was reported to have a saturation magnetization of 50 emu/g at room temperature [54].

**Figure 1:**
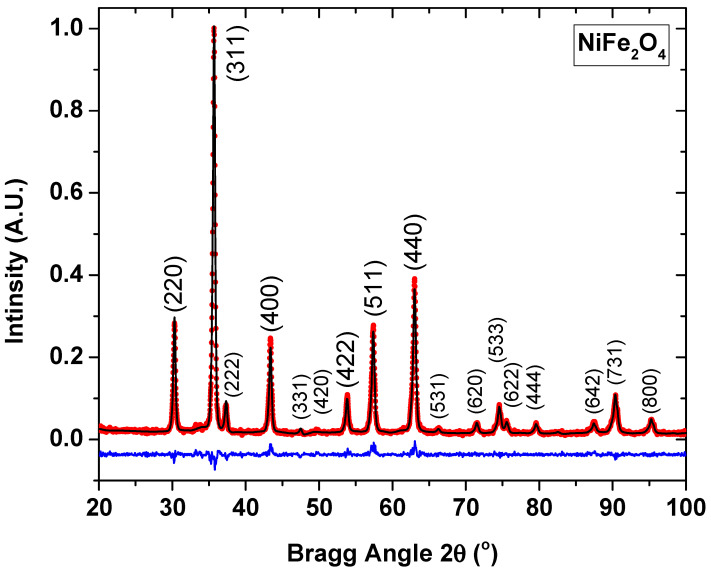
Rietveld refinement of the X-ray diffraction pattern of NiFe_2_O_4_-NPs.

The X-ray diffraction pattern of the prepared nickel ferrite sample (using Cu–*K_α_* radiation with *λ* = 0.15406 nm) is shown in Figure 1. The pattern revealed the presence of a single NiFe_2_O_4_ cubic (fcc) phase consistent with the NiFe_2_O_4_ standard pattern (JCPDS: 10-0325). The characteristic reflections of this phase in Figure 1 were labeled by the corresponding Miller indices (*hkl*). The d-spacing between a given set of parallel atomic planes in the crystal lattice is related to a diffraction peak at angular position (2*θ*) by Bragg’s law:

(1) 2dsinθ=λ

The relation between the lattice constant (*a*) of a cubic crystal and the d-spacing corresponding to a given peak with Miller indices (*hkl*) is rather simple:

(2) $$d=\frac{a}{\sqrt{h^{2}+k^{2}+l^{2}}}$$

The pattern was refined using Rieveld analysis, which revealed the single-phase nature of the sample, and a good fit between the theoretical pattern (the continuous black line) and the experimental pattern (red circles) with a horizontal line representing the difference curve (continuous blue line). The refined lattice parameter was found to be: *a* = (8.347 ± 0.007) Å. This value is in good agreement with the value of 8.34 Å reported for bulk Ni ferrite [54].

**Figure 2:**
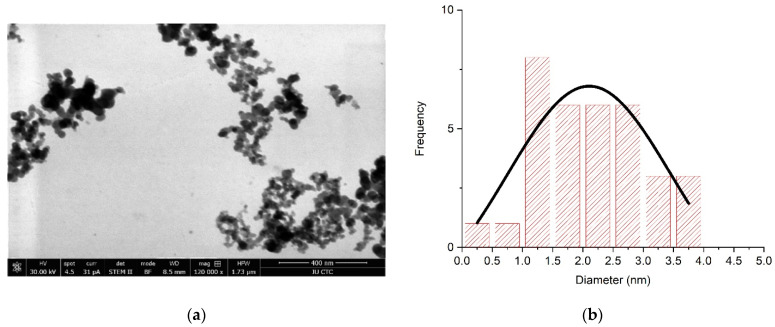
(**a**) TEM image of the NiFe_2_O_4_-NP and (**b**) particle size distribution with a Gaussian fit. Fit $y=y_{0}+\frac{A}{w\sqrt{\frac{\pi }{2}}}e^{-2{\left(\frac{x-x_{c}}{w}\right)}^{2}}$ with *R*^2^ = 0.67, where *y*_0_ = −2.677 ± 18.510, *x_c_* = 2.105 ± 0.213, *w* = 2.712 ± 4.025, and *A* = 32.186 ± 10.782.

**Figure 3:**
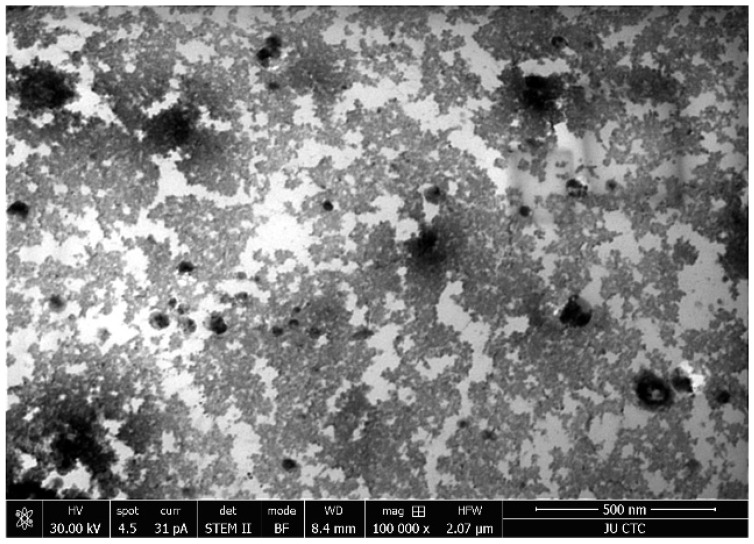
Field emission scanning electron microscopy (FESEM) image of the NiFe_2_O_4_-NPs.

Based on the values we obtained from the TEM, the size of the synthesized particles was 2.5–5 nm. The structure and morphology of the synthesized nanoparticles are shown in the FESEM and TEM images (Figure 2 and Figure 3). The particle size distribution obtained from the TEM image was dominated by particle sizes between 2 and 5 nm (Figure 2b).

### 3.2. Thermo Gravimetric Analysis (TGA)

**Figure 4:**
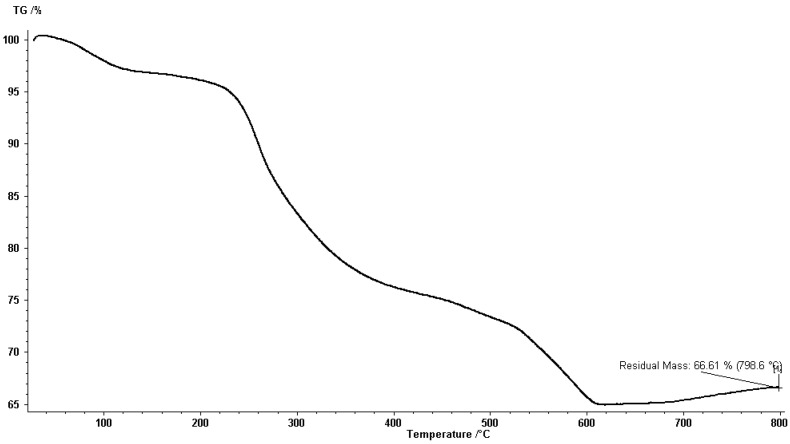
Thermogravimetric analysis (TGA) curve of NiFe_2_O_4_-NPs.

The TGA curve consists of the following three stages (Figure 4). The first stage (temperature range 50–150 °C) is due to the initial breakdown of the complex and evaporation of the absorbed water. The spontaneous combustion is caused by interactions of organic compounds in the plant extract gel with liberation of H_2_O, CO_2_, and some NO_x_ [25,26,27]. The second region (250–500 °C) is attributed to further oxidation of the organic matter and decomposition of inorganic salts, which has two degradation mechanisms involving both intramolecular and intermolecular transfer reactions [28,29,30,31]. The third stage (600–800 °C) is due to the formation of the corresponding metal oxide. When the temperature was increased beyond 640 °C, no weight loss was observed, indicating that NiFe_2_O_4_ oxide was formed.

### 3.3. Magnetization

**Figure 5:**
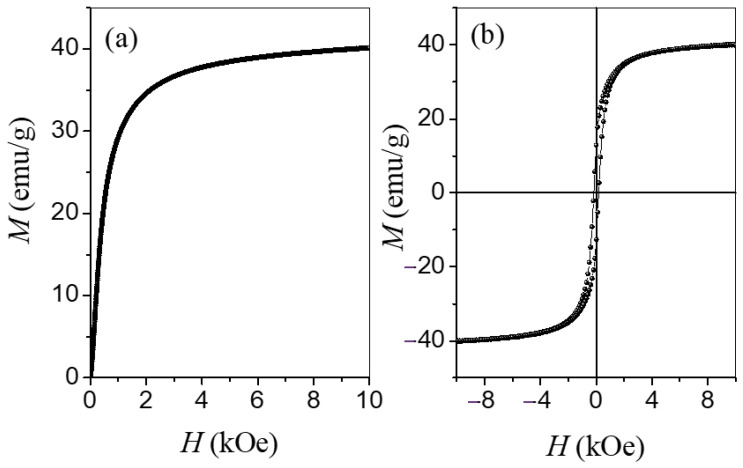
(**a**) Initial magnetization curve and (**b**) hysteresis loop of the NiFe_2_O_4_ spinel ferrites.

Important magnetic parameters of the nickel ferrite nanoparticles were determined from measurements of the initial magnetization curve (Figure 5a) and of the hysteresis loop (Figure 5b). The magnetization in Figure 5a initially increased sharply with the increase of the applied field intensity (*H*), and then exhibited an approach to saturation behavior at high fields above 2 kOe. The magnetic hysteresis loop in Figure 5b revealed a low coercivity (*H_c_*), confirming the soft magnetic character of the sample. Analysis of the magnetization behavior in the high filed and low field ranges allowed for the determination of important properties of the material.

Specifically, the relation *M* vs. 1/*H* at high fields (*H* > 8.5 kOe) is linear, and extrapolation of the straight line to 1/*H* = 0 gives an intrinsic property of the ferrite, namely, the saturation magnetization (*M_s_*). On the other hand, the slope of the straight line can be used to determine the smallest particle diameter (*D_s_*) in the particle size distribution, whereas the largest particle diameter in the distribution (*D_l_*) can be determined from the initial (low field) magnetic susceptibility [56,57]. The coercivity (*H_c_*) of the ferrite was determined from the hysteresis loop (Figure 5b). The parameters determined from the initial magnetization curve and the hysteresis loop are listed in Table 1.

The saturation magnetization of 42.2 emu/g was lower than the value of 50 emu/g reported for bulk nickel ferrite [54], but higher than values reported for nickel ferrites exhibiting superparamagnetic behavior [58,59]. The large variations of the saturation magnetization could be associated with phase purity, and the particle size of the ferrite phase, being lower for smaller particles due to the existence of a spin-glass surface layer, and the increase of surface-to-core fraction with the decrease of particle diameter. The calculated magnetic particle diameter was in the range 9.0–11.1 nm, indicating a sharp particle size distribution. This size, however, is a measure of the magnetic core diameter of the particle. The hysteresis loop in Figure 5b indicated that the coercivity (*H_c_*) of the sample was 180 Oe. The non-vanishing coercivity is an indication that the particle diameter is larger than the critical superparamagnetic diameter. Following the analysis of the magnetic data in [56], the magnetic anisotropy constant (*K*) of the nickel ferrite was estimated to be 7.84 × 10^5^ erg/cm^3^ (from Equation (6) in [56]). Accordingly, the critical superparamagnetic volume (*V_c_* at room temperature) was calculated using the relation *KV_c_*/*k_B_T* ≅ 4.5 [60], from which the critical superparamagnetic diameter (*D_c_*) was estimated to be 7.8 nm. This result is an indication that the range of particle diameters in the sample was above the critical superparamagnetic diameter, which explains the non-vanishing coercivity of the sample.

**Figure 6:**
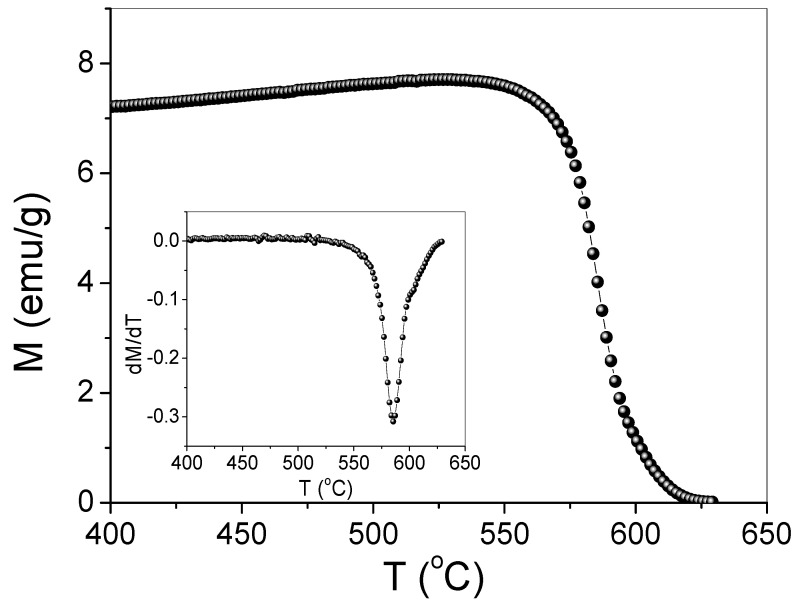
Thermomagnetic curve of NiFe_2_O_4_-NPs in an applied field of 100 Oe (the inset is the derivative of the thermomagnetic data).

To further characterize the synthesized nickel ferrite, the magnetization versus temperature measurements (thermomagnetic measurements) were carried out in an applied magnetic field of 100 Oe, and the results are shown in Figure 6. The curve shows a single steep decline of the magnetization down to zero at the ferromagnetic-paramagnetic transition temperature (Curie temperature, *T_c_*). The Curie temperature was estimated from the position of the negative peak of the derivative curve (inset of Figure 6), and found to be 585 °C, which is the value reported for NiFe_2_O_4_ spinel ferrite [54].

**Figure 7:**
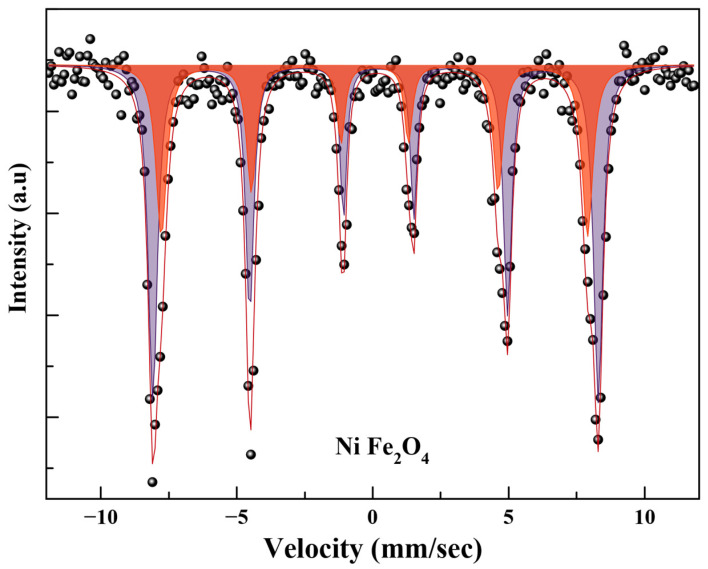
Mössbauer spectrum of spinel NiFe_2_O_4_-NPs.

The Mössbauer spectrum of the Ni-ferrite (Figure 7) clearly shows a magnetically split spectrum, indicating magnetically ordered spins of the iron ions, which is consistent with the magnetic results. The asymmetric absorption lines indicate that the iron ions occupy sites with different chemical environments. The spectrum was best fitted with two magnetic sextets, corresponding to iron ions at tetrahedral and octahedral sites. The hyperfine fields (491 and 513 kOe) and center shifts (0.18 and 0.27 mm/s) of the tetrahedral and octahedral components, respectively, indicate that iron ions were Fe^3+^ in both sites [58]. The observed relative intensities of 38:62 for the tetrahedral-to-octahedral sub-spectra indicate that the sample was a mixed spinel, with a degree of inversion of 0.24. Accordingly, the distribution of cations in the sample was consistent with the formula (Fe_0.76_Ni_0.24_)[Fe_1.24_Ni_0.76_]O_4_, where the round brackets refer to the tetrahedral (A) site, and the square brackets refer to the octahedral [B] site. The distribution of iron and divalent nickel ions at the tetrahedral site was shown to depend on the pH value of the solutions used to prepare Ni-rich spinel ferrites, and the observed distribution is in agreement with that observed in Ni-rich ferrites prepared at pH = 8 [58].

### 3.4. Catalytic Oxidation and Optimization

**Scheme 1:**
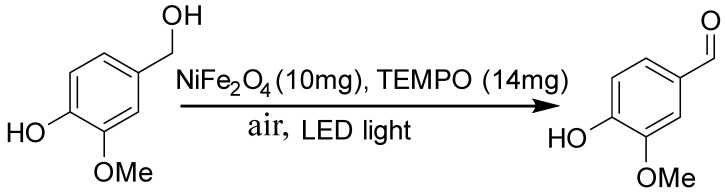
Photocatalytic oxidation of vanillyl alcohol using NiFe_2_O_4_-NPs.

We studied the catalytic properties of NiFe_2_O_4_-NP and its oxidation reactivity towards valorization of lignin using VAL alcohol as model with respect to light intensity, base type, solvent, time, and TEMPO amount (Scheme 1).

**Figure 8:**
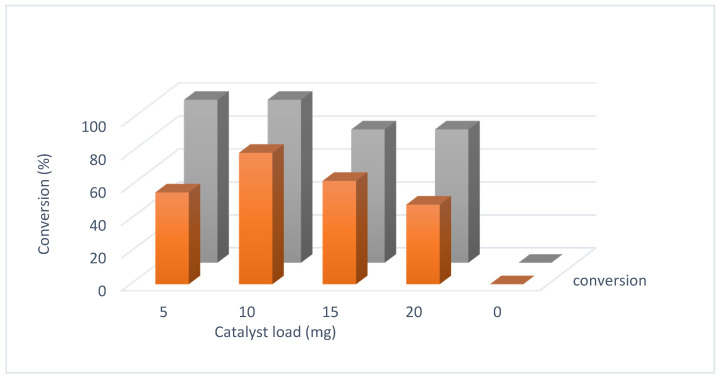
The effect of the amount of catalyst on the oxidation of VAL using NiFe_2_O_4_.

**Figure 9:**
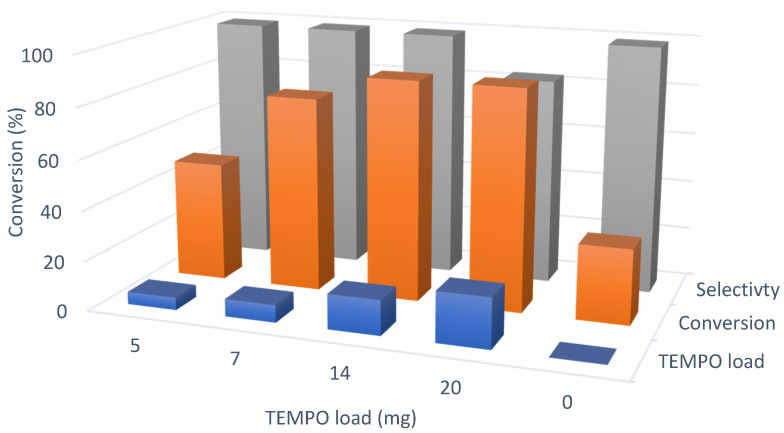
Optimization of the reaction parameters for the oxidation of vanillyl alcohol (VAL) to vanillin (VN) with respect to the TEMPO load. Reaction conditions: 1 mmol vanillyl alcohol, 0.4 mol.% NiFe_2_O_4_, air, rt, 10 mL ACN, base 2 equiv., and 6 h reaction time. Conversion was determined by GC using 1,2-dichlorobenzene as the internal standard.

**Figure 10:**
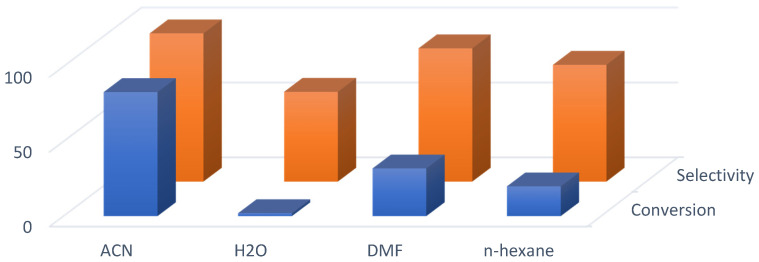
Optimization of the reaction parameters for the oxidation of VAL to VN with respect to the solvent type. Reaction conditions: 1 mmol vanillyl alcohol, 0.4 mol.% NiFe_2_O_4_, air, rt, 10 mL solvent, 0.8 mol.% TEMPO, base 2 equiv., and 6 h reaction time. Conversion was determined by GC analysis using 1,2-dichlorobenzene as the internal standard.

**Figure 11:**
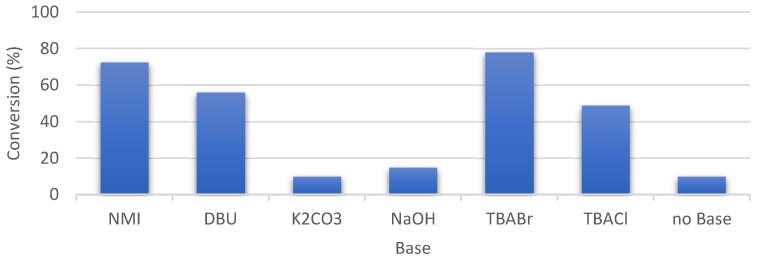
Optimization of the reaction parameters for the oxidation of VAL to VN with respect to the base type. Reaction conditions: 1 mmol vanillyl alcohol, 0.4 mol.% NiFe_2_O_4_, air, rt, 10 mL solvent, 0.8 mol.% TEMPO, base 2 equiv., and 6 h reaction time. Conversion was determined by GC analysis using 1,2-dichlorobenzene as the internal standard.

**Figure 12:**
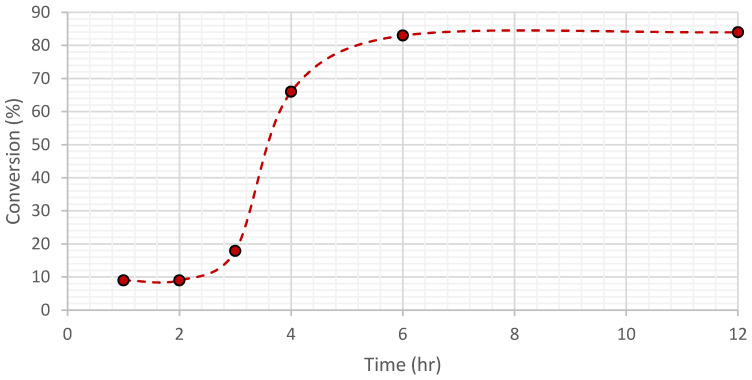
Effect of irradiation time on the oxidation of VAL using NiFe_2_O_4_.

**Figure 13:**
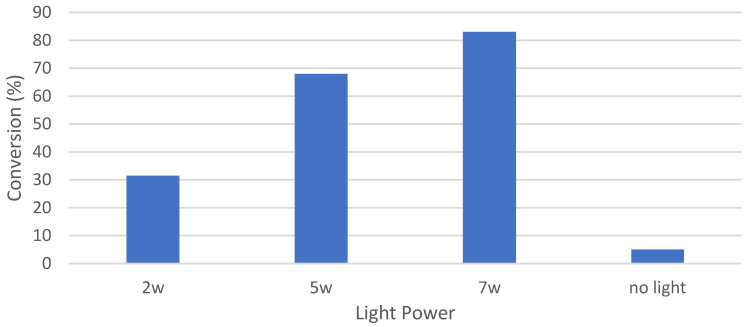
Optimization of light power for the oxidation of vanillyl alcohol to VN. Reaction conditions: 1 mmol vanillyl alcohol, 0.4 mol% NiFe_2_O_4_, air, rt, 10mL solvent, 0.8 mol.% TEMPO, base 2 equiv., and 6 h reaction time. Conversion was determined by GC analysis using 1,2-dichlorobenzene as the internal standard.

The optimization was performed with respect to the catalyst loading (0, 5, 10, 15, and 20 mg; Figure 8), TEMPO amount (0, 5, 7, 14, and 20 mg; Figure 9), solvent type (ACN, H_2_O, DMF, and n-hexane; Figure 10), base type (NMI, DBU, K_2_CO_3_, NaOH, NH_4_Br, NH_4_Cl, and no base; Figure 11), light irradiation time (1, 2, 3, 4, 6, and 12 h; Figure 12), and power (2, 5, and 7 w; Figure 13). The optimization was done with one parameter at a time, while all other parameters were kept fixed.

When the amount of catalyst was increased from 5 to 10 mg (Figure 8), the conversion of VAL toward VN increased from 56% to 83%, with 99% selectivity in both cases. When the amount of catalyst was increased to 15 or 20 mg, both the conversion and the selectivity decreased, as vanillic acid was detected by GC. In the absence of the catalyst, no product formation was observed.

When the TEMPO amount was increased from 5 to 7 mg (Figure 9), the conversion of VAL increased from 49% to 78%, with 99% selectivity of the main product in both cases. When the TEMPO amount was increased to 14 mg, the conversion was improved with 88.5% and 99.9% selectivity to VN. Upon a further increase of the TEMPO amount to 20 mg, the conversion remained the same, but the selectivity of VN decreased to 82% due to the formation of vanillic acid as a byproduct. In the absence of TEMPO the VN formation was noted, but in low yield, indicating that TEMPO has a significant role in the reaction mechanism via stabilizing the radical OH which contributes to the formation of the aldehyde.

The conversion and selectivity of VN was the highest when acetonitrile ACN was used as a solvent (Figure 10). Although water is more polar than ACN, the conversion and selectivity decreased to 69% and 64%, respectively, when using water. Lower conversion and selectivity were observed when n-hexane and DMF were used.

The base’s effect on the reaction of VAL was tested by adding 2 equiv. of the base (Figure 11). The increase of conversion from 10 to 89% was obtained when tetrabutylammonium bromide (TBABr) salt was used with selectivity to VN up to 99%; the reaction reached high turn over number (TON) and turn over frequency (TOF) values of 1466 and 244 h^−1^, respectively. A control experiment with no base added to the reaction gave only 15% conversion with no selectivity for vanillin, which shows the importance of the base as a component in this protocol.

When the irradiation time was 2 h, the conversion of VAL was 14% (Figure 12). By prolonging the irradiation time to 6 h, the conversion to VN increased to 83%. When the reaction time was prolonged to 12 h, there was no increase in the VN conversion and the selectivity decreased. This was due to acid formation and other by-products obtained, such as aldol condensation. Therefore, 6 h was the optimum reaction time to obtain the highest reactivity and selectivity for VN. Upon applying LED light, the reactivity was about five times more than in the absence of light (Figure 13). When the LED’s power was 7 W, the photocatalytic reaction had its highest rate and about 80% VN was produced. The light intensity has an effect on the photocatalytic reaction via exciting the valence electrons to the conduction band [61,62,63,64,65].

Not only does light influence photocatalytic oxidation, but solvent type does too. Acetonitrile addition was previously reported to promote the formation of active radical species [63,64,65]. The formation of active oxygen species, such as superoxide radicals, was proposed to be a leading pathway for the formation of aldehyde. Further comprehensive mechanistic study is ongoing.

After optimization, the catalyst showed high reactivity and high selectivity toward vanillin. For instance, we obtained VN at 89% yield under benign conditions. Through a simple regeneration technique, the catalysts were reused several times. After having the optimization condition in hand, we tested the scope of the catalyst for the oxidation of another lignin substrate models, such as veratryl alcohol, cinnamyl alcohol, and furfaryl alcohol; we obtained the aldehyde product with 84–90% yield (Table 2 entry 2, 3 and 4).

### 3.5. Comparative Studies

We compared our results to previously reported heterogenous systems for VAL oxidation (Table 3). Most of these catalysts have converted VAL into vanillin with 24–92.6% yields under highly basic or acidic solutions, and through a simple regeneration technique the catalysts were reused several times. In this work, the catalyst showed high reactivity and high selectivity toward vanillin. Interestingly, until now no studies have used visible-light-induced photocatalysis, TEMPO, and aerobic conditions in lignin substrate model oxidation.

### 3.6. Recyclability

A unique property of heterogeneous catalyst is its recyclability [66,67,68,69,70]. Therefore, we investigated the recyclability by using VAL as a substrate in the presence of 10 mg of the catalyst under the optimized conditions.

**Figure 14:**
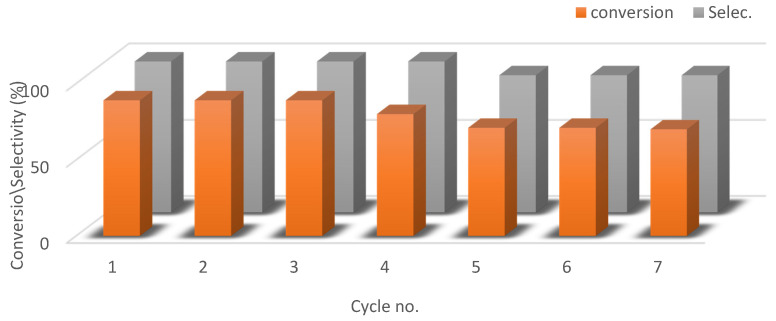
Recyclability test of the NiFe_2_O_4_-NP catalyst. The weight loss of catalyst after four runs was 25 wt.%.

The catalyst was recycled for up to three successive runs without loss in activity. The catalyst was washed with water and successively with ethanol before it was reused for the oxidation of VAL. (Figure 14). Interestingly, after the 4th run the weight loss of the catalyst was 20 wt.% using gravimetric assay. The drop in the reactivity can be due to slight deactivation of the catalyst via leaching some of the metals in the nanoparticles; however, after the fifth cycle the conversion and the selectivity remained constant.

## 4. Conclusions

In this study, we report a phyto-mediated Np of NiFe_2_O_4_ using *Boswellia carterii* extract. The NiFe_2_O_3_ catalyst was prepared by mixing equivimolar Ni(NO_3_)_2_ and FeCl_3_ in an aqueous plant extract that was pH controlled, followed by calcination at 800 °C. The applicability of the method as a method of green synthesis of nanoparticles is adequate, as the aqueous plant extract acts as a reducing agent and prevents coagulation, which keeps the particle size small. The magnetic NiFe_2_O_4_ nanoparticles was characterized by powder XRD, TGA, FESEM, HRTEM, Mössbauer spectroscopy, and VSM measurements. The powder XRD and the FESEM results indicated a spinel ferrite with an average diameter of 2–5 nm. The material showed high catalytic activity, selectivity, and turn over number (TON) for valorization of lignin substrate (VAL)—89%, 99%, and 1466 respectively. The catalytic system used mild reaction conditions, such LED light, air as oxidant, and ACN as solvent. The addition of bases such TBABr and TEMPO to the system led to improving the catalytic reactivity. This improvement in the catalytic reactivity could have been due to stabilization of the OH radical, which plays an important role in the reaction mechanism. Interestingly, TEMPO alone showed no catalytic reactivity in our reaction conditions.

This protocol, with its magnetic nature and its predictable selectivity, was applied for other lignin models such as veratryl, cinnamyl, and furfaryl alcohols; and to our delight, it showed high reactivity and selectivity toward the aldehyde products. The magnetic NPs catalytic system is easily obtained and reused up to four times without a drastic change in the reactivity; the reduction in the reactivity on the fifth use would be due to leaching of metals. Interestingly, after the fifth cycle, the reactivity and the selectivity remain the same up to the seventh cycle.

## Figures and Tables

**Table 1:** Saturation magnetization (*M_s_*), coercivity (*H_c_*), particle diameters, and magnetic anisotropy constant (*K*) for the NiFe_2_O_4_ sample.

*M_s_* (emu/g)	*H_c_* (Oe)	*D_s_* (nm)	*D_l_* (nm)	*K* (erg/cm^3^)	*D_c_* (nm)
42.2	180	9.0	11.1	7.84 × 10^5^	7.8

**Table 2:** Lignin-based oxidation of different substrates.

**Entry**	**Substrate**	**Yield (Ald. Selectivity) (%) ^1^**
1	Vanillyl alcohol	88 (99)
2	Veratryl alcohol	90 (99)
3	Cinnamyl alcohol	84 (95)
4	Furfaryl alcohol	85 (99)

^1^ Using optimized conditions and 6 h reaction time. Yield was isolated yield.

**Table 3:** Vanillyl alcohol oxidation comparative studies.

Entry	Catalyst	Conditions	Main Products	Yield (%)	Refrences
1	CoTiO_4_/H_2_O_2_	298 K, MeOH, CH_3_COOH, 6 h	VN, Vanillic acid	68 and 31	[61]
2	Co_3_O_4_	413 K, 0.689–4 MPa, H_2_O, 7 h	Vanillic acid, VN	69 and 9	[62]
3	TEMPO@SiO_2_/Cu/air	303 K, IPA, NMI, 1 h	VN	92.6	[65]
4	2.3%TiO_2_/fluorescent lamps	353 K, NaOH pH 13	VN	44	[66]
5	Au–Pd@HT visible light, air	298 K, dioxan, 24 h	VN	52.8	[47]
6	NiFe_2_O_4_/TEMPO/visible light, air	298 K, TBABr, airACN, 12 h	VN	89	This work
